# Everybody nose: molecular and clinical characteristics of nasal colonization during active methicillin-resistant *Staphylococcus aureus* bloodstream infection

**DOI:** 10.1186/s12879-022-07371-w

**Published:** 2022-04-24

**Authors:** Erika Reategui Schwarz, Adriana van de Guchte, Amy C. Dupper, Ana Berbel Caban, Devika Nadkarni, Lindsey Fox, Alexandra Mills, Ajay Obla, Kieran I. Chacko, Irina Oussenko, Flora Samaroo, Jose Polanco, Richard Silvera, Melissa L. Smith, Gopi Patel, Melissa Gitman, Bremy Alburquerque, Marilyn Chung, Mitchell J. Sullivan, Harm van Bakel, Deena R. Altman

**Affiliations:** 1 Division of Infectious Diseases, Department of Medicine, Icahn School of Medicine at Mount Sinai, New York City, NY USA; 2 Department of Genetics and Genomic Sciences, Icahn School of Medicine at Mount Sinai, New York City, NY USA; 3 Icahn Institute for Data Science and Genomic Technology, Icahn School of Medicine at Mount Sinai, New York City, NY USA; 4 Department of Pathology, Icahn School of Medicine at Mount Sinai, New York City, NY USA

**Keywords:** Methicillin-resistant *Staphylococcus aureus*, Bloodstream infections, Colonization, Molecular epidemiology

## Abstract

**Background:**

Healthcare-associated infections pose a potentially fatal threat to patients worldwide and *Staphylococcus aureus* is one of the most common causes of healthcare-associated infections. *S. aureus* is a common commensal pathogen and a frequent cause of bacteremia, with studies demonstrating that nasal and blood isolates from single patients match more than 80% of the time. Here we report on a contemporary collection of colonizing isolates from those with methicillin-resistant *S. aureus* (MRSA) bloodstream infections to evaluate the diversity within hosts, and detail the clinical features associated with concomitant nasal colonization.

**Methods:**

Swabs of the bilateral anterior nares were obtained from patients diagnosed with MRSA bacteremia. A single colony culture from the blood and an average of 6 colonies from the nares were evaluated for MRSA growth. For the nares cultures, we typed multiple isolates for staphylococcal protein A (*spa*) and derived the clonal complexes. Demographic and clinical data were obtained retrospectively from the electronic medical record system and analysed using univariate and multivariable regression models.

**Results:**

Over an 11-month period, 68 patients were diagnosed with MRSA bloodstream infection, 53 were swabbed, and 37 (70%) were colonized with MRSA in the anterior nares. We performed molecular typing on 213 nasal colonies. *Spa* types and clonal complexes found in the blood were also detected in the nares in 95% of the cases. We also found that 11% of patients carried more than one clone of MRSA in the nares. Male sex and history of prior hospitalization within the past 90 days increased odds for MRSA colonization.

**Conclusion:**

The molecular epidemiological landscape of colonization in the setting of invasive disease is diverse and defining the interplay between colonization and invasive disease is critical to combating invasive MRSA disease.

**Supplementary Information:**

The online version contains supplementary material available at 10.1186/s12879-022-07371-w.

## Background

*Staphylococcus aureus* (*S. aureus*) is one of the most common causes of healthcare-associated infections (HAI), associated with high mortality rates in invasive disease [1, 2]. *S. aureus* is also a common commensal pathogen and approximately 20–36% of the human population may be intermittently or persistently colonized, often asymptomatically [3–5]. Although *S. aureus* can colonize all skin surfaces, the most frequently assessed site of colonization is the anterior nares [6]. Nasal carriage plays a key role in progression onward to invasive disease [7], and persistent carriers have higher relative risk to develop bacteremia compared to non-carriers [8].

Within-host diversity of nasal carriage with *S. aureus* has been estimated to be 6.6% [9], and people commonly become infected with one of their colonizing isolates [10]. In a multicenter study, 82% of patients with MRSA bacteremia had identical clones in the anterior nares, and 86% of patients with MRSA colonization in anterior nares had clonally identical isolates later obtained from blood [10]. Colonization is also key to subtle transmission events and has been a central feature of outbreaks [11, 12]. In the United States, most *S. aureus* infections are caused by two dominant clonal complexes (CC), CC5 and CC8 [2]. CC5 has historically encompassed hospital-associated genotypes, and CC8 community genotypes. Our work [13] and that of others [14, 15] have found clonal clinical correlations are merging, with classic community genotypes becoming a common cause of hospital-associated infections. Here, we tested the associations between infection with dominant MRSA genotypes and the salient clinical features for the study population.

Given the importance of *S. aureus* colonization in invasive disease, we investigated the molecular epidemiological landscape of patients with MRSA bloodstream infections (BSI) with concomitant nasal colonization. Our aim was to collect a contemporary set of colonizing isolates from those with MRSA BSI, evaluate the frequency of diversity in the colonizing isolates, and define clinical features associated with the presence of colonization. We hypothesized that for patients with active MRSA BSI, there would be clinical differences between those concomitantly colonized and those who were not, as the presence of colonization itself is an important marker of disease status. We thus examined the clinical features and outcomes of patients with active MRSA BSI both with and without concomitant nasal colonization. Further definition of the link between colonization and infection will enable future avenues for mitigation of this infection.

## Results

### Molecular diversity of nasal colonization in those with MRSA BSI

**Fig. 1:**
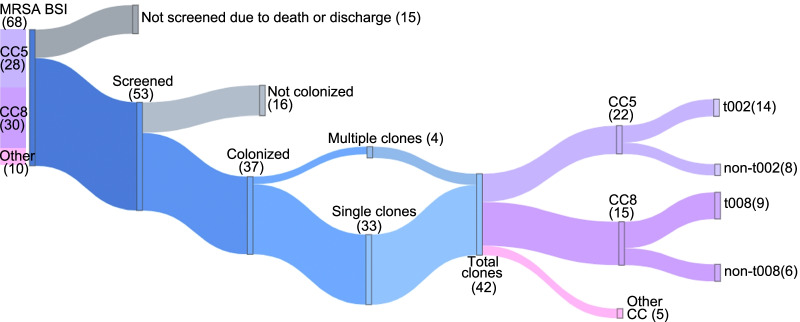
Sankey flow chart diagram of 68 patients with MRSA BSI detailing nasal colonization status with breakdown by *spa *and clonal complex (CC)

Over an 11-month period (July 2018 to May 2019), 68 unique patients were diagnosed with MRSA BSI. Sequencing of single colonies from the blood cultures revealed that 30 (44%) were caused by CC8, 28 (41%) by CC5 and 10 (15%) by other clonal complexes (Fig. 1). From the 68 patients with MRSA BSI, 53 were successfully swabbed in the nares and screened for MRSA colonization. Fifteen patients were excluded due to discharge or death at the time of identification. From the 53 individuals who underwent nasal swabbing, 37 (70%) were colonized with MRSA. The average number of colonies picked from the nasal growth plates was 6 (range 2–12), resulting in a total of 213 which underwent *spa* typing (Additional file 1: Table S1). It was found that 95% of patients had at least one matching *spa* type between the blood isolate and nasal isolate. Four of the 37 colonized patients (11%) carried multiple *spa* types in the anterior nares, resulting in a total of 42 distinct nasal clones. Among the individuals who had swabs positive for MRSA, 22 of the nasal *spa* types corresponded to CC5 and 15 corresponded to CC8, and 5 with other CCs (Fig. 1). Within the CC5 samples, t002 represented the most common *spa* type, present in 14 of the 22 samples (64%), while t008 presented as the most common *spa* type amongst the CC8 samples, in 9 of the 15 samples (60%) (Additional file 1: Table S1). Four MRSA blood isolates were also identified as novel sequence types (ST); ST6964, ST6983, ST7090, ST7094.

### Epidemiological and clinical characteristics associated with bacteremia and colonization

The 68 patients with MRSA BSI were predominantly male (n = 41, 60%), between the age of 55 and 69 (n = 28; 41%). The patients were 34% (n = 23) non-Hispanic white, 34% (n = 23) non-Hispanic black, 28% (n = 19) Hispanic, and 4% (n = 3) belonged to other races or ethnicity groups (Table 1). Patients were more frequently admitted from home (n = 46; 68%), but more than half of the patients had a history of previous hospitalization within the prior 90 days (n = 42; 62%). Community-onset (CO) infections, or infections which present within the first 3 days of hospitalization, were more common than hospital-onset (HO) (n = 40; 59% vs n = 28; 41%) infections, however 75% (n = 51) of patients had an invasive device in place at the time of infection. A prior personal history of colonization, as defined as any prior positive culture for MRSA from any site, or a documented history of prior MRSA infection or colonization, was present 28% of the time. The sources of infection were predominantly deemed to be unknown/other (32%), vascular access (22%), and skin and soft tissue (21%), followed by peripheral IV (10%), and pneumonia (7%) (Table 1).

**Table 1:** Demographics and clinical characteristics of patients with MRSA BSI

Factor, n (%)	(*n* = 68)
Sex	
Male	41 (60)
Female	27 (40)
Race/ethnicity	
Non-hispanic white	23 (34)
Non-hispanic black	23 (34)
Hispanic/latinos	19 (28)
Asian	2 (3)
Other/not reported	1 (1)
Age at time of infection	
18–54 years	18 (26)
55–69 years	28 (41)
≥ 70 years	22 (32)
History of IV drug use	4 (6)
HIV	7 (10)
Admission source	
Home^a^	46 (68)
NH/Rehab/LTACH	15 (22)
Outside hospital	7 (10)
Prior hospital admission (90 days)	42 (62)
Length of hospital stay prior to BSI	
CO-MRSA	40 (59)
HO-MRSA	28 (41)
Clonal complex	
CC5	28 (41)
CC8	30 (44)
Other	10 (15)
Frequent healthcare interaction	
Hemodialysis	14 (21)
Infusion center^b^	14 (21)
None	40 (59)
Presence of invasive device^c^	51 (75)
Invasive procedures (missing = 1)^d^	25 (37)
Wound present^e^	35 (51)
Comorbidities^f^	
Myocardial infarction	11 (16)
Congestive heart failure	18 (26)
Peripheral vascular disease	11 (16)
Cerebrovascular disease	11 (16)
Dementia	8 (12)
Chronic pulmonary disease	15 (22)
Connective tissue disease	3 (4)
Peptic ulcer disease	1 (1)
Mild liver disease	4 (6)
Diabetes (no complications)	18 (26)
Diabetes with organ damage	11 (16)
Hemi or paraplegia	7 (10)
Moderate/severe renal disease	14 (21)
Solid tumor	2 (3)
Leukemia	1 (1)
Lymphoma/multiple myeloma	10 (15)
Moderate/severe liver disease	5 (7)
Metastatic solid tumor	11 (16)
Charlson comorbidity index (CCI)	
0–3	13 (19)
4–5	20 (29)
6–8	22 (32)
> 8	13 (19)
History of transplant^g^	8 (12)
History of MRSA colonization^h^	19 (28)
Presumed source of MRSA BSI	
Peripheral IV	7 (10)
Skin and soft tissue infection	14 (21)
Pneumonia	5 (7)
Diabetic foot infection	1 (1)
Vascular access	15 (22)
Sacral wound	4 (6)
Other/unknown source^i^	22 (32)

*IV* intravenous, *NH* nursing home, *Rehab* rehabilitation facility, *LTACH* long-term acute care hospital, *CO* community-onset, *HO* hospital-onset, *MRSA* methicillin-resistant *Staphylococcus aureus*, *BSI* bloodstream infection, *HIV* human immunodeficiency virus

^a^ “Admission from home”: included nonmedical residences such as home, group homes, assisted living facilities, and homeless shelters

^b^“Infusion center”: outpatient centers for chemotherapy, intravenous fluids, intravenous immunomodulators, and blood products

^c^“Presence of invasive device”: included pacemaker, implantable cardioverter defibrillator (ICD), left ventricular assist device (LVAD), vascular access (excluding peripheral intravenous catheters), orthopedic hardware, nephrostomy, suprapubic catheter, ileal conduit, foley catheter, arteriovenous graft placement (AVG), percutaneous endoscopic gastrostomy (PEG) tube, or ostomy

^d^“Invasive procedures”: included any invasive procedures or surgery occurring within 1 month before first positive blood culture for MRSA, excluding electroencephalogram (EEG), electrocardiogram (EKG), and transthoracic echocardiogram (TTE)

^e^“Wound present”: presence of a chronic skin wound overlying the sacrum, limb, abdomen, or other body part

^f^“Comorbidities”: as defined by the Charlson Comorbidity Index (CCI); refer to standard definitions for CCI

^g^“History of transplant”: included solid organ and bone marrow transplant

^h^“History of MRSA colonization”: any positive culture from urine, sputum, tissue, or nares with MRSA prior to the positive MRSA blood culture or a documented history of prior MRSA infection or colonization

^i^“Other/Unknown source”: MRSA infection from urinary source, osteomyelitis, surgical site infection, spinal infection, septic arthritis or cardiac device infection, or an unknown/not reported source

Multivariable analysis found that those with concomitant MRSA colonization were more likely to be male, comprising 73% (n = 27) of the colonized patients compared to 44% (n = 7) in the non-colonized group (OR, 5.06; 95% CI, 1.64–22.01; *p* = 0.03) (Table 2). Patients were more likely to have a prior hospital admission within 90 days (n = 26; 70%) compared to the non-colonized (n = 6; 38%) (OR, 3.96; 95% CI, 1.01–15.56; *p* = 0.05). Patients with a history of renal disease including need for hemodialysis were more likely to be colonized (OR, 15.52; 95% CI, 1.13–213.37; *p* = 0.04), while dementia was negatively associated with colonization (OR, 0.10; 95% CI, 0.01–1.02; *p* = 0.05) (Additional file 2: Table S2). There were no significant differences in outcomes between the two groups (Additional file 3: Table S3). All patients who underwent swabbing were treated with standard of care anti-MRSA therapy (intravenous vancomycin), and there was no significant difference between the time of bacteremia and the time of nasal swab acquisition between colonized and non-colonized patients (Wilcoxon rank sum t-test *p* = 0.1738). Patients were not actively decolonized with anti-MRSA antibiotics, such as intranasal mupirocin.

**Table 2:** Demographics and clinical characteristics of patients with MRSA BSI with and without nasal colonization

Factor	Colonized N = 37 (%)	Not colonized N = 16 (%)	Univariate analysis OR (95% CI)	*p* value	Multivariable analysis OR (95% CI)	*p* value
Sex						
Male	27 (73)	7 (44)	**3.47 (1.02–11.82)**	**0.05**	**5.06 (1.64–22.01)**	**0.03**
Female	10 (27)	9 (56)	Reference		Reference	
Race/ethnicity						
Non-hispanic white	13 (35)	7 (44)	Reference			
Non-hispanic black	11 (30)	4 (25)	1.48 (0.34–6.43)	0.60		
Hispanic/latino	11 (30)	5 (31)	1.19 (0.29–4.81)	0.81		
Asian	1 (3)	0 (0)	–	–		
Other/not reported	1 (3)	0 (0)	–	–		
Age at time of infection						
18–54 years	12 (32)	2 (13)	Reference			
55–69 years	16 (43)	6 (38)	0.44 (0.08–2.60)	0.37		
≥ 70 years	9 (24)	8 (50)	0.19 (0.03–1.11)	0.06		
History of IV drug use						
Yes	2 (5)	1 (6)	0.86 (0.07–10.19)	0.90		
No	35 (95)	15 (94)	Reference			
HIV diagnosis						
Yes	6 (16)	0 (0)	–	–		
No	31 (84)	16 (100)	–	–		
Admission source						
Home	22 (59)	13 (81)	Reference			
NH/Rehab/LTACH	10 (27)	2 (13)	2.96 (0.56–15.63)	0.20		
Other hospital	5 (14)	1 (6)	2.96 (0.31–28.14)	0.35		
Prior hospital admission (90 days)						
Yes	26 (70)	6 (38)	**3.94 (1.15–13.52)**	**0.03**	**3.96 (1.01–15.56)**	**0.05**
No	11 (30)	10 (63)	Reference		Reference	
Length of hospital stay prior to BSI						
CO-MRSA	20 (54)	10 (63)	Reference			
HO-MRSA	17 (46)	6 (38)	1.42 (0.43–4.71)	0.57		
Clonal complex						
CC8	13 (35)	9 (56)	Reference			
CC5	19 (51)	5 (31)	2.63 (0.72–9.66)	0.15		
Other	5 (14)	2 (13)	1.73 (0.27–10.97)	0.56		
Frequent healthcare interaction						
Hemodialysis	12 (32)	1 (6)	8.00 (0.92–69.84)	0.06		
Infusion Center	7 (19)	3 (19)	1.56 (0.33–7.24)	0.57		
None	18 (49)	12 (75)	Reference			
Presence of invasive device						
Yes	30 (81)	10 (63)	2.57 (0.70–9.48)	0.16		
No	7 (19)	6 (38)	Reference			
Invasive procedures						
Yes	13 (35)	4 (25)	1.63 (0.44–6.07)	0.47		
No	24 (65)	12 (75)	Reference			
Wound present						
Yes	21 (57)	6 (38)	2.19 (0.66–7.29)	0.20		
No	16 (43)	10 (63)	Reference			
Charlson comorbidity index (CCI)						
0–3	4 (11)	5 (31)	Reference		Reference	
4–5	12 (32)	4 (25)	3.75 (0.66–21.25)	0.14	7.44 (0.95–58.45)	0.06
6–8	15 (41)	4 (25)	4.69 (0.84–26.08)	0.08	**7.84 (1.03–59.84)**	**0.05**
> 8	6 (16)	3 (19)	2.50 (0.37–16.89)	0.35	3.68 (0.44–30.88)	0.23
History of transplant						
Yes	6 (16)	1 (6)	2.90 (0.32–26.33)	0.34		
No	31 (84)	15 (94)	Reference			
History of MRSA colonization						
Yes	14 (38)	2 (13)	4.26 (0.84–21.60)	0.08		
No	23 (62)	14 (88)	Reference			
Presumed source of MRSA BSI						
Peripheral IV	5 (14)	2 (13)	1.09 (0.19–6.33)	0.92		
Skin and soft tissue infection	7 (19)	4 (25)	0.70 (0.17–2.84)	0.62		
Pneumonia	3 (8)	1 (6)	1.32 (0.13–13.78)	0.81		
Diabetic foot infection	1 (3)	0 (0)	–	–		
Vascular access	11 (30)	3 (19)	1.83 (0.43–7.74)	0.41		
Sacral wound	2 (5)	2 (13)	0.40 (0.05–3.13)	0.38		
Other/unknown	8 (22)	4 (25)	0.83 (0.21–3.28)	0.79		

See Table 1 for definitions

Bold = significant at ≤ 0.05

*IV* intravenous, *NH* nursing home, *Rehab* rehabilitation facility, *LTACH* long-term acute care hospital, *CO* community-onset, *HO* hospital-onset, *MRSA* methicillin-resistant *Staphylococcus aureus*, *BSI* bloodstream infection, *HIV* human immunodeficiency virus

When comparing clinical characteristics associated with two predominant clonal complexes (CC5 and CC8) in patients bacteremic during the entire study period, admission from a rehabilitation facility was significantly more common in those infected with CC5 (n = 11; 39%) compared to CC8 (n = 4; 13%) (OR, 3.78; CI 1.02–14.06; *p* = 0.05) (Table 3). Furthermore, patients in the CC5 group had significantly higher rates of hospitalizations within the prior 90 days (n = 22; 79%) compared to those with CC8 (n = 15; 50%) (OR, 3.67; CI 1.16–11.60; *p* = 0.03). In the multivariable analysis, the presence of an invasive device was 93% (n = 26) in the CC5 group and 60% (n = 18) in the CC8 group (OR, 9.75; CI 1.87–50.85; *p* = 0.007). Although not approaching significance, the primary source of BSI due to CC8 vs. CC5 was 13% vs 5% in skin derived infections (due to either peripheral IV or skin and soft tissue infection). While the 30-day mortality rate for all bacteremic patients was 26%, there were no differences in mortality and outcomes between those infected with either CC8 or CC5.

**Table 3:** Demographics and clinical characteristics of patients with MRSA BSI due to CC5 vs. CC8

Factor	CC5 N = 28 (%)	CC8 N = 30 (%)	Univariate analysis OR (95% CI)	*p* value	Multivariable analysis OR (95% CI)	*p* value
Sex						
Male	20 (71)	16 (53)	2.19 (0.74–6.50)	0.16	2.63 (0.80–8.61)	0.11
Female	8 (29)	14 (47)	Reference		Reference	
Race/ethnicity						
Non-hispanic white	10 (36)	11 (37)	Reference			
Non-hispanic black	11 (39)	8 (27)	1.51 (0.43–5.28)	0.52		
Hispanic/latino	7 (25)	9 (30)	0.86 (0.23–3.16)	0.82		
Asian	0 (0)	1 (3)	–	–		
Other/not reported	0 (0)	1 (3)	–	–		
Age at time of infection						
18–54 years	7 (25)	8 (27)	Reference			
55–69 years	9 (32)	15 (50)	0.69 (0.19–2.54)	0.57		
≥ 70 years	12 (43)	7 (23)	1.96 (0.49–7.77)	0.34		
History of IV drug use						
Yes	0 (0)	3 (10)	–	–		
No	28 (100)	27 (90)	–	–		
HIV diagnosis						
Yes	3 (11)	3 (10)	1.08 (0.20–5.86)	0.93		
No	25 (89)	27 (90)	Reference			
Admission source						
Home	16 (57)	22 (73)	Reference			
NH/Rehab/LTACH	11 (39)	4 (13)	**3.78 (1.02–14.06)**	**0.05**		
Other hospital	1 (4)	4 (13)	0.34 (0.04–3.37)	0.36		
Prior hospital admission (90 days)						
Yes	22 (79)	15 (50)	**3.67 (1.16–11.60)**	**0.03**		
No	6 (21)	15 (50)	Reference			
Length of hospital stay prior to BSI						
CO-MRSA	17 (61)	19 (63)	Reference			
HO-MRSA	11 (39)	11 (37)	1.12 (0.39–3.23)	0.84		
Frequent healthcare interaction						
Hemodialysis	9 (32)	4 (13)	3.00 (0.77–11.62)	0.11		
Infusion center	4 (14)	6 (20)	0.89 (0.21–3.72)	0.87		
None	15 (54)	20 (67)	Reference			
Presence of invasive device						
Yes	26 (93)	18 (60)	**8.67 (1.73–43.49)**	**0.009**	**9.75 (1.87–50.85)**	**0.007**
No	2 (7)	12 (40)	Reference			
Invasive procedures (missing = 1)						
Yes	10 (36)	11 (37)	0.91 (0.31–2.67)	0.86		
No	18 (64)	18 (60)	Reference			
Wound present						
Yes	18 (64)	12 (40)	2.70 (0.93–7.82)	0.07		
No	10 (36)	18 (60)	Reference			
Charlson comorbidity index (CCI)						
0–3	5 (18)	7 (23)	Reference			
4–5	9 (32)	8 (27)	1.58 (0.35–7.00)	0.55		
6–8	11 (39)	8 (27)	1.93 (0.45–8.33)	0.38		
> 8	3 (11)	7 (23)	0.60 (0.10–3.54)	0.57		
History of transplant						
Yes	4 (14)	1 (3)	4.83 (0.51–46.18)	0.17		
No	24 (86)	29 (97)	Reference			
History of MRSA colonization						
Yes	9 (32)	5 (17)	2.37 (0.68–8.23)	0.17		
No	19 (68)	25 (83)	Reference			
Presumed source of MRSA BSI						
Peripheral IV	1 (4)	5 (17)	0.19 (0.02–1.70)	0.14		
Skin and soft tissue infection	4 (14)	8 (27)	0.46 (0.12–1.74)	0.25		
Pneumonia	2 (7)	1 (3)	2.23 (0.19–26.06)	0.52		
Diabetic foot infection	1 (4)	0 (0)	–	–		
Vascular access	8 (29)	6 (20)	1.60 (0.48–5.38)	0.45		
Sacral wound	4 (14)	0 (0)	–	–		
other/unknown	8 (29)	10 (33)	0.80 (0.26–2.45)	0.70		

See Table 1 for definitions

Bold = significant at ≤ 0.05

*IV* intravenous, *NH* nursing home, *Rehab* rehabilitation facility, *LTACH* long-term acute care hospital, *CO* community-onset, *HO* hospital-onset, *MRSA* methicillin-resistant *Staphylococcus aureus*, *BSI* bloodstream infection, *HIV* human immunodeficiency virus

## Methods

### Study setting, patient identification, and isolate selection

The Mount Sinai Hospital (MSH) is a quaternary care facility. Under the approval of the MSH institutional review board (HS # 18-00662; 13-00981), adults (≥ 18 years old) with MRSA BSI were identified by the MSH Clinical Microbiology Laboratory as part of standard clinical care between July 2018 to May 2019. Identification and susceptibility of MRSA was performed by the Clinical Microbiology Laboratory using VITEK^®^2 (bioMerieux). Per hospital policy, all patients with MRSA BSI are placed on contact isolation with staff donning gown and gloves for interactions. Swabbers washed their hands prior to donning gown and gloves, and additionally wore surgical masks during sample collection. Bilateral anterior nares were sampled using a single, pre-labeled, sterile swab (Puritan^®^ PurFlock^®^ Ultra Sterile Flocked Collection Devices) moisturized with sterile 0.9% NaCl saline solution inserted up to 2.5 cm from the edge of the nares and rolled 5 times in each nostril. Swabs were plated directly on CHROMID^®^ MRSA plates. After 24 h of incubation at 37 °C, the plate was evaluated for positive MRSA growth. If positive for MRSA, the isolate lawns were collected and used to inoculate Tryptic Soy Broth (TSB) with 15% glycerol cryovials. If negative for growth, the plates were re-evaluated at 48 h. When needed, Staphaurex™ Latex Agglutination Test was used to confirm coagulase positivity. The cryovial contents were homogenized using a Vortex-Genie 2 and then stored in a − 80 °C freezer.

For the molecular work up, the frozen stocks from the nasal swab stocks were plated on BD BBL™ Trypticase™ Soy Agar (TSA II™) with Sheep Blood and incubated at 37 °C. Selection of colonies was performed using a previously described replica plating technique [16] in which nasal swab stocks were diluted in 1X PBS and spread onto TSA II plates before incubation for 16 h at 37 °C to facilitate an even spread of distinct colonies. From these base plates we replica plated onto new TSA II Blood plate, BD BBL™ Prepared Plated Media: Mannitol Salt Agar, CHROMID^®^ MRSA plates, and BD BBL™ Oxacillin Screen Agar (Mueller Hinton Agar w/6 µg/mL Oxacillin, 4% NaCl) Prepared Media plates, respectively. These replica plates were grown at 37 °C for 24 h before examination for phenotypic differences in colony morphology. The aim was to select approximately 6 (ideally morphologically diverse) colonies (Additional file 1: Table S1) for every swab sample, and bacterial stocks were prepared using TSB with 15% glycerol. All colonies were analysed using a Bruker MALDI Biotyper^®^ System to confirm the growth of *S. aureus.*

### Molecular typing

Single colonies from the clinical MRSA blood cultures underwent DNA extraction and PacBio whole genome sequencing as part of a surveillance program, as described previously [11]. From the resulting genomes a custom script (https://github.com/mjsull/spa_typing) was run to identify the *spa* type. The raw sequence data for the blood genomes have been deposited in the National Center for Biotechnology Information SRA database under Bioproject PRJNA470993.

DNA extraction of the nasal cultures was performed on a ThermoFisher KingFisher Flex 96 using Applied BioSystems MagMAX™ DNA Multi-Sample Ultra 2.0 Kits. Typing of the *spa* gene was performed by first using PCR to amplify the *spa* sequence through use of previously described methods [17], and then Sanger sequenced by Psomagen Inc. The Lasergene software package (version 17, DNAstar) was used to generate consensus sequences for *spa* and ST typing. Assignment of new STs was performed by submission of the DNA sequences of seven housekeeping genes (*arcC*, *aroE*, *glpF*, *gmk*, *pta*, *tpi*, and *yqiL*) to the online MLST database https://pubmlst.org/ [18]

### Patient data collection and statistical analyses

Under approval obtained from the Mount Sinai Hospital Institutional Review Board (HS# 17-00825), demographic and clinical data were obtained retrospectively from the electronic medical record system, including admission data, presumed source of the BSI based on Infectious Diseases (ID) consults, comorbidities, and prior outpatient health care exposures. Chart abstraction and data entries were placed into the REDCap [19] system and verified by two different ID trained individuals. All patients diagnosed with MRSA BSI received an ID consultation at the time of diagnosis, as per standard practice at our institution. Surveillance definitions included hospital-onset MRSA (HO-MRSA), defined as positive cultures on or after the fourth day after hospital admission, and community-onset MRSA (CO-MRSA), defined as BSI presenting within the 72-h hospital admission interval [20]. We also evaluated in-hospital outcomes and death, especially those considered to be related to the MRSA BSI. We categorized all non–normally distributed continuous variables into discrete categorical groups. The univariate logistic regression model was performed initially, and variables with *p* values ≤ 0.2 were placed in the multivariable logistic regression model. All variables with *p* ≤ 0.05 were considered statistically significant. All analyses were performed in SAS (version 9.4) [21].

## Discussion

Colonization is an important feature of invasive MRSA disease. In our study we found that 70% of patients with MRSA BSI were colonized with MRSA in the anterior nares. In 95% of these cases, the MRSA clones present in the blood were also found in the nares, suggesting infection from an endogenous source.

As nasal colonization was associated with history of a recent hospitalization within the prior 90 days, presumably these individuals may become colonized with exposure to the healthcare setting. While baseline colonization is not assessed on admission at our hospital, nasal colonization with MRSA has been independently associated with healthcare exposure in prior studies [22]. The relationship between recent hospitalization and colonization was implicated through the higher rates of CC5 amongst those with prior healthcare exposures and higher overall rates of colonization with CC5. This may signal differences in the avenues in which BSI due to CC5 and CC8 are acquired given the association of CC5 and the healthcare setting. Additionally, a higher proportion of male patients with MRSA BSI were found to be colonized when compared to women. This finding is consistent with prior studies and may be attributed to differences in the microbiome of the skin such as sweat, sebum, and hormone production along with differences in behavior and hand-hygiene practices [23, 24].

The overall genetic diversity found among the nasal clones was 11%. However, within-patient nasal cultures exhibited a high degree of relatedness with one another, with most clustering into the same genetic clades. A prior study focused on nasal carriage clonality estimated a 6.6% diversity [9], while one pediatric study found 30% diversity over longitudinal time points [25], suggesting that carriage of discordant *S. aureus* clones in individuals with nasal colonization occur regularly and may lead to horizontal genetic exchange among clones. Nonetheless, there is a paucity of data regarding *S. aureus* diversity in nasal colonization. With evaluations limited to a small number of colonies, the actual diversity is likely greater [26].

There were few overall differences in the clinical characteristics of patients infected with either CC5 or CC8 MRSA, upholding prior findings that indicate that historical hospital and community associations of clones are indeed blurred in endemic regions. Skin and soft tissue and peripheral intravenous catheter (PIV) infections tended to correlate more frequently to the CC8 MRSA. While CC8 is associated with community skin derived infections [27], we have previously observed that CC8 was also associated with PIV infections [13] highlighting the importance of community clones causing hospital-onset infections. Colonization was associated with patients with prior history of renal disease, which is a well-established clinical association given the frequent interaction with healthcare facilities and likelihood of requiring vascular access [28].

There are some limitations in this study. The patient population at our institution is complex and might not represent the same population in smaller community hospitals. Regional and global differences in the prevalence of MRSA colonization and clonal types may limit generalizability. The retrospective chart review portion may be subject to errors in chart abstraction. Although we expect that the numerically dominant clones are most likely to be selected, particularly in the setting of bacteremia, we are likely underrepresenting the full diversity [29]. While it is understood that people become infected with their colonizing isolate, we are unable to directly address directionality, as there may have been a single exposure that led to acquisition at both sites. Additionally, exposure to intravenous anti-MRSA antibiotics and/or choice of anti-MRSA antibiotics was not assessed and may have differed between colonized and non-colonized groups. Finally, we focused on MRSA-BSI due to the focus of our molecular surveillance program although we recognize that methicillin-susceptible *S. aureus* (MSSA) also causes significant disease [30].

## Conclusions

Colonization is an essential component of invasive MRSA disease, and we found high rates of colonization, and colonization with more than one clone at a frequency of 11%. This contemporary sample collection adds to the paucity of literature on the frequency and the diversity of colonization in the presence of active MRSA BSI. We found colonization to be associated with male sex and those with a recent hospitalization, highlighting potential high-risk groups. Combined molecular and clinical analyses will assist in defining the intra-host and inter-host dynamics of MRSA and enable the development of targeted approaches to curtail disease.

## Supplementary Information

**Additional file 1: Table S1**. MRSA isolate typing.

**Additional file 2: Table S2**. Comorbidities of patients with MRSA BSI with and without nasal colonization.

**Additional file 3: Table S3**. Outcomes of patients with MRSA BSI with and without nasal colonization.

## Data Availability

The raw sequence data for the blood genomes that support the findings of this study have been deposited in the National Center for Biotechnology Information SRA database under Bioproject PRJNA470993; other data used and/or analysed during the current study are available from the corresponding author on reasonable request.

## Abbreviations

HAI: Healthcare-associated infections
MRSA: Methicillin-resistant *S. aureus*
BSI: Bloodstream infections
*spa*: Staphylococcal protein A
*S.** aureus*: *Staphylococcus aureus*
CC: Clonal complex
CO: Community-onset
HO: Hospital-onset
MSH: Mount Sinai Hospital
TSB: Tryptic Soy Broth
TSA II™: Trypticase™ Soy Agar
ID: Infectious diseases
HO-MRSA: Hospital-onset MRSA
CO-MRSA: Community-onset MRSA
ST: Sequence type
MSSA: Methicillin-susceptible *S. aureus*
