# Supplementary material for: Everybody nose: molecular and clinical characteristics of nasal colonization during active methicillin-resistant Staphylococcus aureus bloodstream infection
Source: BMC Infect Dis. 2022 Apr 24;22:400. doi: 10.1186/s12879-022-07371-w (PMC9036699; doi:10.1186/s12879-022-07371-w)
Supplement: Supplementary file 2 — Additional file 2: Table S2. Comorbidities of patients with MRSA BSI with and without nasal colonization. [file 12879_2022_7371_MOESM2_ESM.docx]

**Supplementary Table 2. Comorbidities of patients with MRSA BSI with and without nasal colonization**

| **Comorbidities** | **Colonized**  **N = 37 (%)** | **Not**  **Colonized**  **N = 16 (%)** | **Univariate**  **Analysis**  **OR (95% CI) *p* value** | | **Multivariable Analysis**  **OR (95% CI) *p* value** | |
| --- | --- | --- | --- | --- | --- | --- |
| Myocardial Infarction | 9 (24) | 0 (0) | -- | -- |  |  |
| Congestive Heart Failure | 13 (35) | 0 (0) | -- | -- |  |  |
| Peripheral Vascular Disease | 7 (19) | 2 (13) | 1.63 (0.30-8.89) | 0.57 |  |  |
| Cerebrovascular Disease | 6 (16) | 3 (19) | 0.84 (0.18-3.87) | 0.82 |  |  |
| Dementia | 3 (8) | 4 (25) | 0.27 (0.05-1.36) | 0.11 | **0.10 (0.01-1.02)** | **0.05** |
| Chronic Pulmonary Disease | 11 (30) | 3 (19) | 1.83 (0.43-7.74) | 0.41 |  |  |
| Connective Tissue Disease | 1 (3) | 1 (6) | 0.42 (0.02-7.11) | 0.55 |  |  |
| Peptic Ulcer Disease | 1 (3) | 0 (0) | -- | -- |  |  |
| Mild Liver Disease | 2 (5) | 1 (6) | 0.86 (0.07-10.19) | 0.90 |  |  |
| Diabetes (no complications) | 10 (27) | 4 (25) | 1.11 (0.29-4.26) | 0.88 |  |  |
| Diabetes with Organ Damage | 8 (22) | 2 (13) | 1.93 (0.36-10.32) | 0.44 |  |  |
| Para or Hemiplegia | 3 (8) | 1 (6) | 1.32 (0.13-13.78) | 0.81 |  |  |
| Moderate/Severe Renal Disease | 12 (32) | 1 (6) | 7.20 (0.85-61.08) | 0.07 | **15.52 (1.13-213.37)** | **0.04** |
| Solid Tumor | 2 (5) | 0 (0) | -- | -- |  |  |
| Leukemia | 1 (3) | 0 (0) | -- | -- |  |  |
| Lymphoma/Multiple Myeloma | 5 (14) | 3 (19) | 0.68 (0.14-3.25) | 0.63 |  |  |
| Moderate/Severe Liver Disease | 4 (11) | 1 (6) | 1.82 (0.19-17.68) | 0.61 |  |  |
| Metastatic Solid Tumor | 3 (8) | 3 (19) | 0.38 (0.07-2.14) | 0.27 |  |  |

**Bold** = significant at ≤ 0.05
