# Supplementary material for: Everybody nose: molecular and clinical characteristics of nasal colonization during active methicillin-resistant Staphylococcus aureus bloodstream infection
Source: BMC Infect Dis. 2022 Apr 24;22:400. doi: 10.1186/s12879-022-07371-w (PMC9036699; doi:10.1186/s12879-022-07371-w)
Supplement: Supplementary file 3 — Additional file 3: Table S3. Outcomes of patients with MRSA BSI with and without nasal colonization. [file 12879_2022_7371_MOESM3_ESM.docx]

**Supplementary Table 3**. **Outcomes** **of patients** **with MRSA BSI with and without nasal colonization**

| **Outcomes** | | **Colonized**  **N = 37 (%)** | **Not Colonized**  **N = 16 (%)** | **Univariate**  **Analysis**  **OR (95% CI) *p* value** | | |
| --- | --- | --- | --- | --- | --- | --- |
| *30 Day Mortality* | 7 (19) | 1 (6) | 3.50 (0.39-31.09) | | 0.26 | |
| *30 Day Mortality Related to MRSA* | 4 (57) | 0 (0) | -- | -- | | |
| 60 Day Mortality | 9 (24) | 2 (13) | 2.25 (0.43-11.85) | 0.34 | | |
| *60 Day Mortality Related to MRSA* | 4 (44) | 0 (0) | -- | -- | | |
| *90 Day Mortality* | 9 (24) | 3 (19) | 1.39 (0.32-6.01) | 0.66 | | |
| *90 Day Mortality Related to MRSA* | 4 (44) | 0 (0) | -- | -- | | |
| *^a^Recurrent bacteremia* | 9 (24) | 2 (13) | 2.25 (0.43-11.85) | 0.34 | | |
| *Duration of Bacteremia, Median (IQR)* | 2.45 (2.00, 8.24) | 3.30 (1.08-5.67) |  | 0.51 | | |
| *ICU Admission after MRSA BSI* | 23 (62) | 12 (75) | 0.55 (0.15-2.03) | 0.37 | | |
| *^b^Mechanical Ventilation after MRSA BSI* | 30 (81) | 13 (81) | 0.99 (0.22-4.44) | 0.99 | | |
| *^c^Metastatic Infection* | 10 (27) | 6 (38) | 0.62 (0.18-2.14) | 0.45 | | |

Abbreviations: MRSA, methicillin-resistant *Staphylococcus aureus*; ICU, intensive care unit.

^a^ Recurrent bacteremia indicates a newly positive blood culture >30 days after the last positive blood culture.
^b^ Mechanical ventilation excludes patients who were perioperative that are intubated < 4 days.
^c^ Metastatic infection is defined as evidence of bacterial seeding to other body sites after initial bloodstream infection (i.e. endocarditis, spinal infection, septic pulmonary emboli).
